# Evaluation of the truebeam machine performance check (MPC): OBI X‐ray tube alignment procedure

**DOI:** 10.1002/acm2.12445

**Published:** 2018-09-03

**Authors:** Michael P. Barnes, Dennis Pomare, Frederick W. Menk, Buiron Moraro, Peter B. Greer

**Affiliations:** ^1^ Department of Radiation Oncology Calvary Mater Hospital Newcastle Newcastle NSW Australia; ^2^ School of Medical Radiation Sciences University of Newcastle Newcastle NSW Australia; ^3^ School of Mathematical and Physical Sciences University of Newcastle Newcastle NSW Australia; ^4^ Varian Medical Systems Palo Alto CA USA

**Keywords:** machine performance check (MPC), linac quality assurance

## Abstract

Alignment of the On‐Board Imager (OBI) X‐ray tube is important for ensuring imaging to treatment isocenter coincidence, which in turn is important for accurate Image Guided Radiotherapy (IGRT). Varian introduced a new X‐ray tube alignment procedure for the TrueBeam linac in software version 2.5 MR2 as part of the machine performance check (MPC) application. This study evaluated the new procedure against conventional methods and examined the clinical significance of X‐ray tube misalignment. Long term stability and short term repeatability of MPC tube alignment was assessed as well as sensitivity of the method to setup error. Standard quality assurance tests expected to be sensitive to tube misalignment were performed before and after tube alignment. These tests included: IsoCal verification; MPC kV imager offset; Winston‐Lutz: kV imaging to treatment/radiation isocenter coincidence; CBCT image QA using the Catphan phantom; and OBI image geometric accuracy and center pixel alignment. Tube alignment measurements were performed with MPC, the two‐plate method, and wire‐on‐faceplate method. The X‐ray tube was then realigned by approximately 1.01 mm in the tangential plane based upon MPC and the tube alignment and standard quality assurance measurements were repeated. The time taken for each tube alignment method was estimated. The MPC method of tube alignment was found to be repeatable, insignificantly sensitive to phantom setup error and quick and simple to perform. The standard QA tests were generally insensitive to the tube alignment change, possibly because of the IsoCal correction. However, reduction in the magnitude of IsoCal correction and MPC kV imager offset was recorded after tube alignment. There was also apparent improvement in CBCT image uniformity. The MPC procedure is recommended for X‐ray tube alignment.

## 1 INTRODUCTION

The Varian (Varian Medical Systems, Palo Alto, CA, USA) On‐Board Imager (OBI) comprises a kV X‐ray tube and amorphous silicon detector attached to the linac and aligned orthogonal to the treatment beam. The OBI is used to take planar or cone‐beam CT (CBCT) images of the radiotherapy patient so that the patient can be correctly aligned for treatment delivery. This process is called image guided radiotherapy (IGRT) and requires the OBI (kV) imaging isocenter to be coincident with the treatment isocenter.^1^, ^2^, ^3^, ^4^, ^5^, ^6^, ^7^ This is achieved by aligning the OBI (X‐ray source and detector) to be orthogonal to the MV treatment beam, parallel to the plane of gantry rotation and project through the linac isocenter. To achieve the correct X‐ray source positioning the X‐ray tube must be aligned so that: Firstly, The kV‐beam ray that passes through linac isocenter is orthogonal to the (correctly beam steered) MV‐beam central axis and secondly be parallel to the plane of gantry rotation.

On the Varian TrueBeam linac the X‐ray tube is aligned in the factory and the detector panel alignment is calibrated using the IsoCal calibration, which adjusts the detector panel lateral and longitudinal positions as a function of gantry angle to align the DICOM coordinates of the images with treatment isocenter.^7^

With the TrueBeam 2.0 platform Varian released the machine performance check (MPC) application. MPC is a fully integrated image‐based tool for assessing the performance of the TrueBeam critical functions. MPC tests include geometric tests, which utilize a series of kV and 6 MV images of the IsoCal phantom situated in a specific bracket on the IGRT couch top to assess: treatment isocenter size, coincidence of MV and kV isocenters, accuracy of collimator and gantry angles, accuracy of jaw and MLC leaf positions, and accuracy of couch positioning including pitch and roll. All measurements are automated and the user is simply required to set up the IsoCal phantom and bracket onto the treatment couch at position H2 and to beam‐on for each energy. For the geometric tests the system makes all required motions automatically and beams on when everything is in position. Images are automatically analyzed at the TrueBeam console and results are presented. MPC has been evaluated by Clivio et al.^8^ and Barnes and Greer.^9^, ^10^, ^11^ However, in TrueBeam V2.5 Maintenance Release (MR) 2, Varian has added new functionality to MPC including an X‐ray tube alignment procedure. Presently, access to these new features is only available with Varian service Hardware‐Assisted Software Protection (HASP) rights and therefore information on how the new features are measured is not available to the customer.

When MPC is logged in with the Varian HASP key additional results are available with the geometric checks. These results include kV source offset parameters in both axial (inplane) and tangential (crossplane) directions, which are measures of the X‐ray tube alignment. A preset threshold of 1 mm has been set. After the MPC geometric checks are run there is an option to perform tube alignment. When selected a wizard appears directing the user as to which kV source screws need to be adjusted and by how much to correct for the measured kV source offset. Once these adjustments are completed then MPC is run again to verify the adjustment and the IsoCal calibration is required to be updated.

There were three broad aims of the study. The first aim was to evaluate the new MPC method of tube alignment against the current existing methods. This was done in two ways; firstly, the sensitivity of the methods was investigated by taking measurements using all three methods before and after a tube alignment change and secondly, the practicalities of the methods were evaluated qualitatively. Together these provide the reader with information as to which method is superior overall.

The second aim of the study was to assess the clinical significance of tube misalignment so that the reader had context for interpreting the results of the first aim and could make an informed decision about which method to use in their own clinic. This second aim was addressed in a sensitivity experiment whereby the tolerances on standard kV imager QA tests were used as a surrogate for clinical significance. The assessment was then based upon whether a significant, in terms of the accepted test tolerance, change was observed in the standard tests before and after a deliberate change in tube alignment.

The third aim was to investigate the stability and influences on tube alignment or its measurement via MPC. This was done in three ways: Firstly, the short term repeatability of the MPC measurement was assessed. Secondly, the sensitivity of the MPC measurement to setup error was investigated and thirdly, nearly 2‐yr's worth of data on MPC measured tube alignment was provided alongside relevant maintenance events in the same period and it was assessed whether the tube alignment was stable and whether the maintenance events made a significant change to the measured tube alignment. This provides further information to the reader as to when and what they need to consider for tube alignment in their own clinic.

## 2 MATERIALS AND METHODS

### 2.A Materials

Measurements in this study were performed on a single Varian TrueBeam STx linac running software versions 2.0, 2.5 MR1 and later 2.7, and with an aS1200 EPID.

### 2.B Methods

Replacement of the X‐ray tube or other faults can require that the tube be realigned. This has conventionally been performed with either of two methods. The first method, hence known as the two‐plate method, requires that two of the Varian blade calibration plates be aligned with isocenter. The blade calibration plate includes a graticule that is visible in kV images. Tube alignment is typically achieved using firstly one plate placed on the treatment couch at height 20.0 cm below isocenter. The plate is aligned to either the top laser or cross hair with gantry levelled precisely at 0° and an electron cone is placed on top with care not to displace the plate. A second plate is then placed on top of the electron cone and also aligned to laser/cross hair. The linac gantry is then precisely levelled with the OBI kV source pointing to the floor (head up position). An image is taken of the two plates. For correct tube alignment the graticules of the two plates should align in the image. This is assessed qualitatively. The measurement is repeated with OBI source pointed to the ceiling (head down position). The tube alignment is adjusted to achieve best graticule alignment between head up and down.

The second conventional method of OBI X‐ray tube alignment requires only one blade calibration plate setup to isocenter using laser/cross hair. Thin tungsten wires are then carefully attached to the kV source faceplate using the engraved graduations to center the wires in both planes. Images with correct tube alignment show the wires aligned with the blade calibration plate graticule. Similar to the two‐plate method, this is assessed qualitatively. Advantages of this method over the two‐plate method include less reliance on laser/cross hair verticality and no requirement to accurately level the gantry. This latter method shall be henceforth known as the wire‐on‐faceplate method.

To evaluate short term repeatability, MPC was run successively five times and two standard deviations calculated for the kV source offset parameters. Also, an MPC measurement was performed after introducing a deliberate error in the phantom alignment to determine whether the phantom setup affected the measurement. This was achieved by introducing packing between the phantom and its mount to introduce an approximate 2° rotation in the phantom.

To assess the long term stability of the MPC measurements and sensitivity to maintenance activity, nearly 2 yr of daily data was recorded for the kV source offset (tangential and axial) and for the kV imager offset, along with a record of dates where relevant linac maintenance events occurred. This data predated the upgrade to TrueBeam V2.7 and the ability to realign the X‐ray tube using MPC. The mean values and two standard deviations of the kV source offset and kV imager offset data were calculated for each period between maintenance events to assess both the magnitude of the changes caused by the maintenance events and the stability of the X‐ray tube alignment between events according to MPC.

Once the TrueBeam 2.7 upgrade was completed and tube alignment using MPC was available an initial run of the MPC tube alignment procedure was performed. MPC reported that the X‐ray tube was misaligned in the tangential direction by 1.18 mm. Misalignment in the axial direction was measured at 0.09 mm. The MPC threshold for this test is set at 1 mm and hence according to MPC tangential tube realignment was indicated.

Before proceeding to realign the X‐ray tube some preliminary measurements were performed. First, the 6 MV beam that is used in the MPC geometric checks including kV source offset, was checked using the Sun Nuclear (Sun Nuclear Corporation, Melbourne, FL, USA) IC Profiler for both beam angle and beam position steering.^9^ Second, the ceiling laser was checked to project vertically and through treatment isocenter.

After beam steering was verified a series of standard quality assurance tests were performed as a baseline for comparison with post X‐ray tube alignment measurements. These tests included: IsoCal verification; MPC kV imager offset; in‐house Winston Lutz: kV imaging to treatment isocenter coincidence; CBCT image quality based upon the Catphan phantom (The Phantom Laboratory, Salem, NY, USA); and OBI Image geometric accuracy and center pixel alignment to isocenter measurement based upon images of the Varian blade calibration plate phantom. These measurements are representative elements of a standard linac QA program that might be sensitive to OBI X‐ray tube misalignment.

Before adjusting the tube alignment the conventional methods of X‐ray tube alignment were also both performed. These methods were then repeated after tube alignment for comparison to allow the sensitivity of the conventional methods to be compared against MPC. Because the conventional methods are analyzed qualitatively then an assessment of the direction of misalignment indicated was assessed and not the magnitude. A potential source of error when comparing the different methods of tube alignment is the filament size used for the measurements. The Varian X‐ray tubes have a small and a large filament to produce a small and large focal spot size respectively. These focal spots can be slightly misaligned, which could affect the tube alignment results. However, the Varian factory test document for the particular tube being tested included a 0.0 mm misalignment measured between the two filaments and hence the choice of filament size would not affect the tube alignment measurement.

The X‐ray tube was realigned following the MPC procedure, which included updating the IsoCal calibration. MPC was then rerun to get a post adjustment measurement to determine the magnitude of the adjustment according to MPC. The conventional tube alignment methods were also repeated before finally, the standard QA tests were re‐performed to determine whether the tube alignment had any noticeable or meaningful impact on relevant clinically significant linac QA parameters.

At the end of the process the time required to realign the tube using each of the methods was estimated. A precise measurement of the time could not be recorded because only the MPC method was actually used to make the adjustment.

## 3 RESULTS

### 3.A MPC kV source offset short term repeatability

After repeating MPC five successive times the kV source offset was found to be 0.074 ± 0.03 mm (mean ± 2 SD) in the axial (inplane) direction and 0.064 ± 0.033 mm (mean ± 2 SD) in the tangential (crossplane) direction.

### 3.B MPC kV source offset sensitivity to phantom rotation

After introducing a 2° roll in the phantom the MPC kV source offsets were measured to be 0.1 mm in the axial direction and 0.02 mm in the tangential directions. In the axial direction this is within two standard deviations of the repeatability test mean value measured immediately prior. The tangential result is outside two standard deviations.

### 3.C Long term stability and sensitivity to maintenance activities

**Figure 1:**
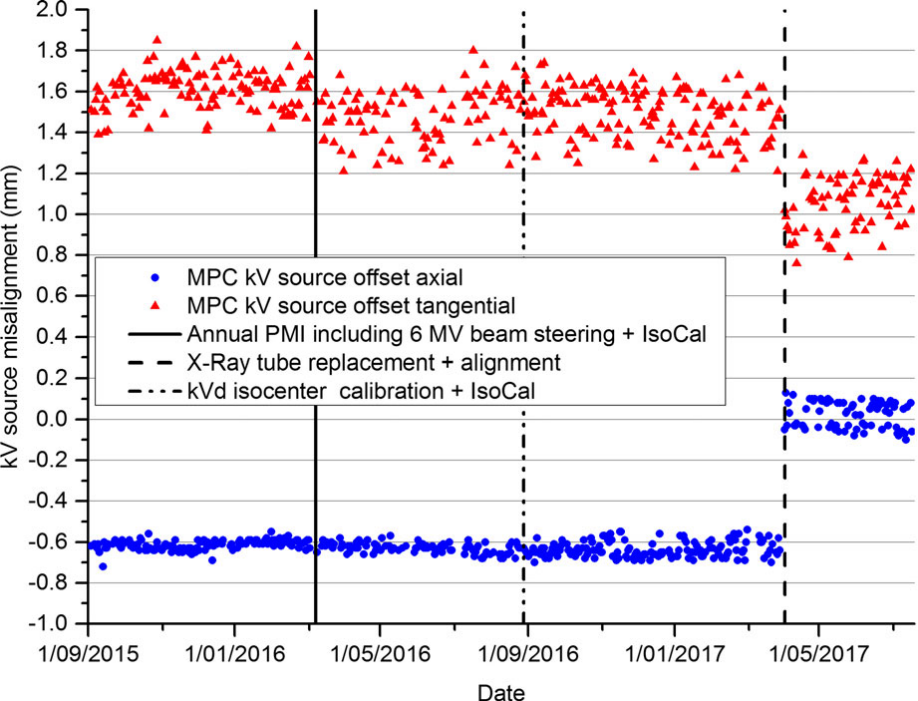
Nearly 2 yrs’ data for MPC kV source offset in axial and tangential directions. Relevant maintenance events are also indicated.

Figure 1 shows the MPC kV source offset results in both axial and tangential directions over a near 2 yr period. The magnitude of the changes in kV source and imager offsets at the maintenance events are presented in Table 1, where the mean and two standard deviations are presented between maintenance events. Vertical lines in Fig. 1 identify times when the X‐ray tube was replaced and aligned, when there was an annual PMI including 6 MV beam steering and IsoCal calibration and when the kVd isocenter calibration was performed with IsoCal calibration. Corresponding changes in the axial (filled circles) and tangential (triangles) source offset are clearly seen. Neither axial nor tangential kV source offset were sensitive to a change in kV detector isocenter calibration, while the tangential offset appears to have changed with Annual PMI: 6 MV beam steering + IsoCal calibration. The mean values before and after this event are within two standard deviations so the results are inconclusive.

**Table 1:** MPC kV imager and source offset results over a 2 yr period presented between relevant maintenance events (mean ± 2 SD (mm))

	kV imager offset	kV source offset	
		Axial	Tangential
Initial to beam steer + IsoCal (*n* = 124)	0.20 ± 0.10	−0.61 ± 0.05	1.61 ± 0.18
Beam steer + IsoCal to kVd isocenter cal (*n* = 92)	0.22 ± 0.07	−0.63 ± 0.05	1.48 ± 0.25
kVd isocenter cal to X‐ray tube replacement (*n* = 141)	0.35 ± 0.07	−0.64 ± 0.08	1.49 ± 0.24
X‐ray tube replacement to end (*n* = 71)	0.17 ± 0.06	0.02 ± 0.13	1.06 ± 0.26

**Figure 2:**
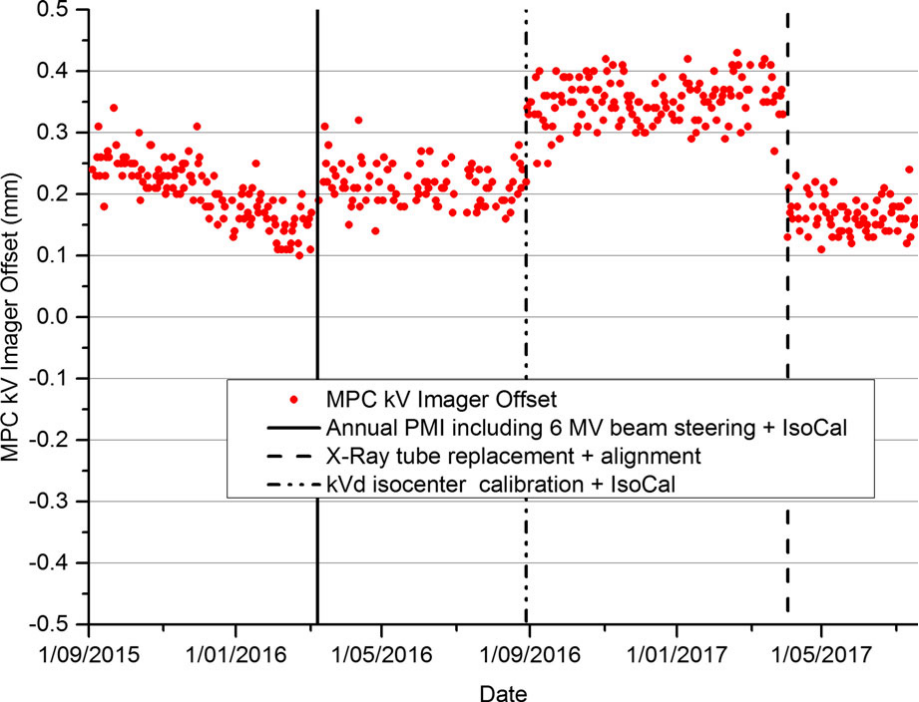
Nearly 2 yr data for MPC kV imager offset. Relevant maintenance events are also indicated.

Figure 2 shows the MPC kV imager offset results over the same near 2 yr period as for Fig. 1. Figure 2 and Table 1 indicates that each of the maintenance events resulted in a change in the kV imager offset result.

### 3.D Initial beam steering

The large field symmetry of the 6 MV beam was measured to be 100.6% and 100.7% for inplane (axial) and crossplane (tangential) respectively using the International Electrotechnical Commission (IEC) definition of symmetry.^12^ The focal spot was found to be aligned to collimator axis (in the isocenter plane) to within 0.1 mm and 0.0 mm for axial and tangential respectively. Both focal spot alignment and large field symmetry were well within departmental beam steering tolerances of 0.4 mm and 1% respectively. Therefore, the 6 MV beam was considered to be sufficiently steered to allow for an accurately aligned 6 MV treatment isocenter for reference for MPC X‐ray tube alignment.

### 3.E Quality assurance tests

#### 3.E.1 IsoCal

The IsoCal verification procedure results performed before and after X‐ray tube alignment are presented in Table 2. The results demonstrate that the realignment of the tube only significantly affected the max imager shift parameter, which was reduced and hence could be considered an improvement.

**Table 2:** IsoCal verification results before and after X‐ray tube alignment

	Before	After	Difference
In‐plane imager rotation kV (deg)	−0.02	−0.018	0.002
Max imager shift kV (mm)	0.44	0.07	−0.37

#### 3.E.2 MPC kV imager offset

Prior to MPC tube realignment the MPC kV imager offset parameter was recorded at 0.4 mm. After adjustment it was measured at 0.63 mm, which is out of tolerance. When the IsoCal calibration was then updated the kV imager offset was measured at 0.13 mm. The results of Table 1 show that this parameter is usually stable with two standard deviations over a recent 6 month period equaling 0.07 mm.

#### 3.E.3 In‐house Winston Lutz

The In‐house Winston Lutz measurements performed before and after X‐ray tube alignment are presented in Table 3. In both measurements the imaging isocenter was checked to be in agreement between full fan and half fan CBCT modes. Both before and after tube realignment the agreement was within 0.1 mm.

**Table 3:** In‐house Winston Lutz results before and after X‐ray tube alignment (coordinates for patient head first and supine)

	Before	After	Difference
3D Isocenter offsets: CBCT vs treatment (mm)			
Left–right	−0.02	−0.16	−0.14
Anterior–posterior	0.03	0.01	−0.02
Superior–inferior	−0.11	−0.04	0.07
2D offsets: treatment field vs CBCT isocenter (mm)			
Gantry maximum displacement	0.40	0.38	−0.02
Gantry average displacement	0.20	0.21	0.01

#### 3.E.4 CBCT Catphan

The CBCT Catphan results for both a full fan mode and a half fan mode measured before and after tube alignment are presented in Table 4. Results are presented as the mean value ± two standard deviations calculated from five successive measurements. The results show no change before and after tube alignment except for the Hounsfield Units (HU) values and image uniformity. The uniformity is improved after the tube alignment.

**Table 4:** CBCT Catphan results before and after X‐ray tube alignment (mean ± 2 SD)

	Full fan		Half fan	
	Before	After	Before	After
Image quality				
Low contrast (disks visible)	1.0 ± 1.1	2.0 ± 1.1	6.0 ± 1.1	6.0 ± 1.1
High contrast (line‐pair patterns discernible)	7.0 ± 0.0	7.0 ± 0.0	5.0 ± 0.0	4.0 ± 0.0
Uniformity (HU)	17.0 ± 5.6	5.0 ± 5.6	10.0 ± 5.6	1.0 ± 5.6
Hounsfield Units (HU)				
Air	−987 ± 2.7	−992 ± 2.7	−999 ± 2.7	−999 ± 2.7
Teflon	983 ± 4.6	1017 ± 4.6	945 ± 4.6	957 ± 4.6
Delrin	350 ± 3.0	375 ± 3.0	350 ± 3.0	357 ± 3.0
Acrylic	117 ± 3.9	132 ± 3.9	118 ± 3.9	121 ± 3.9
Polystyrene	−52 ± 2.0	−41 ± 2.0	−46 ± 2.0	−42 ± 2.0
LDPE	−109 ± 3.7	−93 ± 3.7	−105 ± 3.7	−103 ± 3.7
PMP	−192 ± 9.4	−183 ± 9.4	−195 ± 9.4	−193 ± 9.4
Spatial integrity				
Left–right (mm)	50.1 ± 0.2	50.1 ± 0.2	50 ± 0.2	50 ± 0.2
Anterior–posterior (mm)	50 ± 0.2	49.8 ± 0.2	50.1 ± 0.2	49.9 ± 0.2
Angle (deg)	89.8 ± 0.2	90.2 ± 0.2	90 ± 0.2	90 ± 0.2

#### 3.E.5 OBI geometric checks and center pixel

The results for the OBI geometric image integrity and center pixel for before and after tube alignment are presented in Table 5. The results show no significant change before and after tube alignment.

**Table 5:** OBI geometric integrity and center pixel before and after X‐ray tube alignment

	Before	After	Difference
Image scale			
Left–right (mm)	99.8	99.9	0.1
Superior–inferior (mm)	100	99.9	−0.1
Angle (deg)	90.2	90	−0.2
Center pixel			
Left–right (mm)	0.1	0	−0.1
Superior–inferior (mm)	0.1	0.1	0.0

### 3.F X‐ray tube alignment

**Figure 3:**
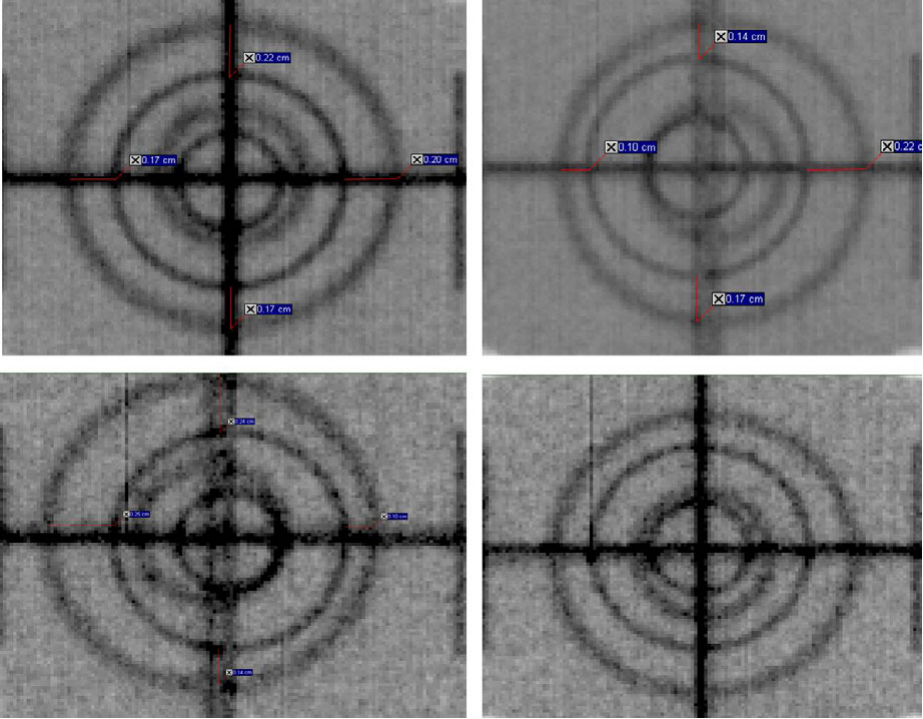
Images for the two‐plate method of X‐ray tube alignment performed before and after tube alignment and at both kV source head up and head down. Top Left = Before and head down, Top Right = Before and head up, Bottom Left = After and head down and Bottom Right = After and head up.

**Figure 4:**
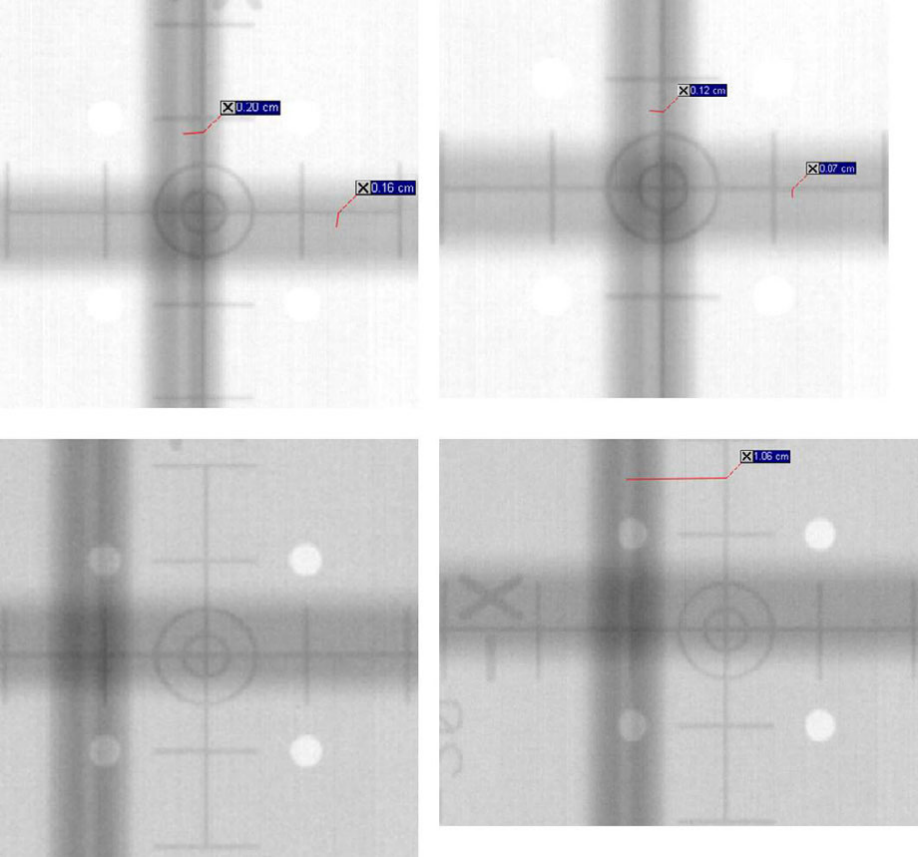
Images for the wires on faceplate method of X‐ray tube alignment performed before and after tube alignment and at both kV source head up and head down. Top Left = Before and head down, Top Right = Before and head up, Bottom Left = After and head down and Bottom Right = After and head up.

Figures 3 and 4 present the images for the two‐plate and wire‐on‐faceplate methods respectively, both before and after X‐ray tube realignment for both kV head up and head down positions.

#### 3.F.1 Before tube realignment

Prior to adjustment of the x‐ray tube alignment the MPC method indicated misalignment of the kV source by +1.18 mm and +0.09 mm in the tangential direction and axial directions, respectively. The 1 mm threshold on this measurement meant that tube realignment was indicated in the tangential direction. For the two‐plate method performed prior to realignment, neither the head up nor down images indicate misalignment in the axial direction. The head up image shows misalignment in the tangential direction while the head down image indicates no tangential misalignment. When averaged, these results would indicate that adjustment is not required in the axial direction, but is required in the tangential direction. Both results are in agreement with MPC.

The images for the wire‐on‐faceplate method before tube realignment indicate misalignment in both axial and tangential directions for the head down orientation and for tangential only for the head up orientation. This would indicate that realignment is required in both planes, although greater in magnitude for the tangential direction. This is in agreement with both MPC and the two‐plate method.

#### 3.F.2 Tube realignment using MPC

**Figure 5:**
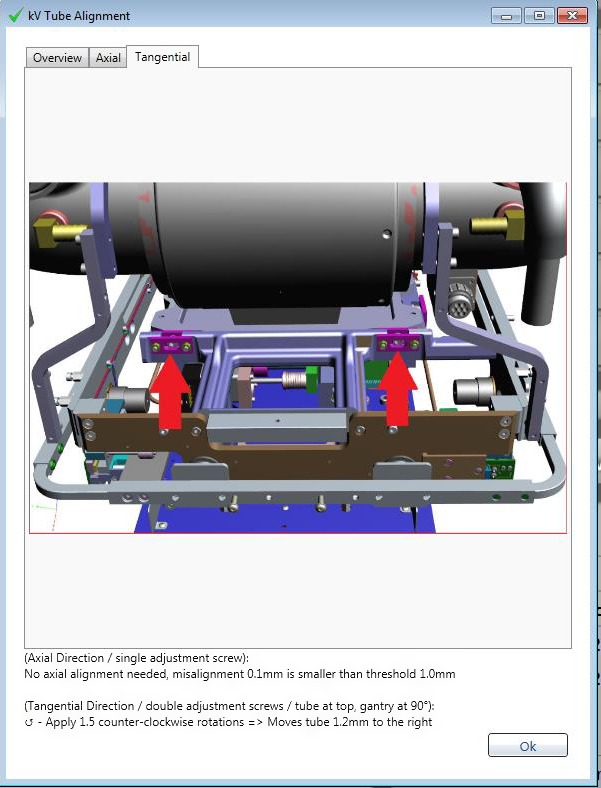
Screen capture of the MPC kV tube alignment procedure in the tangential tab.

To realign the X‐ray tube the MPC method was used. To do this The MPC kV tube alignment feature was chosen and a software wizard appeared. Figure 5 provides a screen capture of this wizard when the tangential tab is selected. The figure shows diagrammatically which screws need adjusting and the embedded text informs how many turns and in which direction the screws need to be turned to make the necessary adjustment. The wizard is unclear in two respects. First, at the points indicated for adjustment there are two opposing screws: one tightens and one loosens. It is not clear that the number of turns indicated applies to both screws. This was determined by trial and error. Second, it was found that there was take‐up slack in the screws before they began to have any influence. It was found through trial and error that the number of turns indicated relates to the situation after the take‐up has been used. As such, the full adjustment was made in two stages.

#### 3.F.3 After tube realignment

When all adjustments had been completed MPC indicated kV source misalignment of −0.17 mm and +0.17 mm for tangential and axial planes respectively. This denotes a change in tube alignment of 1.01 mm and 0.08 mm for tangential and axial planes respectively, indicating an interaction between the axial direction with the tangential direction adjustments. This is potentially introduced when the X‐ray tube mounting bolts were loosened and tightened for the tangential adjustment.

After tube realignment the two‐plate method indicates no misalignment in the axial direction, which agrees with MPC. In the tangential direction the head up image indicates no misalignment while head down indicates misalignment. This is reversed compared to the pre‐realignment images and is not in agreement with MPC.

After tube realignment the wire‐on‐faceplate images show small axial misalignment in both head up and down positions, which within qualitative assessment uncertainty can be considered in agreement with MPC. However, in the tangential direction the wire‐on‐faceplate method indicates greater misalignment in both head up and down positions compared to the pre‐realignment results, which is in disagreement with MPC.

### 3.G Total adjustment time required

Based upon the time required to perform measurements for each of the three tube alignment methods and previous experience with the two‐plates and wire‐on‐faceplate methods, it is estimated that (a) the two‐plate method normally requires approximately 2 h, (b) the wire‐on‐faceplate method takes approximately one and a half hours, and (c) the MPC method takes approximately 45 min.

## 4 DISCUSSION

### 4.A Short term repeatability

The results show that the axial and tangential kV source offset parameters are repeatable to the order of a few hundredths of a millimeter. In this study repeatability is defined as two standard deviations of a set of five consecutive measurements. A few hundredths of a millimeter is very small compared to the 1 mm threshold set in MPC. This indicates that the measurement is sufficiently repeatable to be able to reliably perform its intended function and the variation is insignificant for X‐ray tube alignment.

### 4.B. kV source offset sensitivity to phantom rotation

Since a roll in the phantom effectively shifts the phantom's ball bearings tangentially in the image rather than radially it is expected that the tangential kV source offset would be more sensitive than axial to phantom rotation. The 2° magnitude of the roll used here is extreme and unlikely to occur in practice, since a roll of this magnitude introduced by an error in the roll of the 6° of freedom couch would be detected by the MPC in other tests. The results indicate that the tangential kV source offset parameter is in fact the plane which is sensitive to phantom roll, whereas the axial kV source offset is not. However, the measured change in tangential kV source offset is well within the threshold and considered not significant to the tube alignment procedure, so realistic phantom rolls are unlikely to introduce significant error in the tube alignment procedure.

### 4.C. Long term stability and sensitivity to maintenance

The near 2‐yr kV source offset results presented in Fig. 1 and Table 1 indicates that the tube alignment measurement is more stable in the axial direction than the tangential direction. This may be because the tangential direction is in the plane of gantry rotation and hence components in this plane are subjected to greater variation in the direction in which gravity is acting. The results also show clear sensitivity of the measurement to realignment of the X‐ray tube for a tube replacement. Figure 1 and Table 1 also indicate that within two standard deviations that there was minimal or no effect on the MPC source offset measurement due to 6 MV beam steering + IsoCal calibration or with kV detector isocenter calibration + IsoCal. Since both the kV detector isocenter calibration plus IsoCal work on the kV detector rather than kV source then it is expected that recalibrating them would have no effect on the kV source offset.

The results of Table 1 indicate that within two standard deviations the MPC kV imager offset parameter was sensitive to all of the maintenance events listed except for the annual PMI: Beam steer and IsoCal calibration. However, for this event Fig. 1 seems to indicate a change in trend at this time, which indicates that the kV imager offset was sensitive to this event. Since the IsoCal calibration was updated at each event and the kV imager offset parameter provides a measure of the IsoCal validity then this result is as expected. In the cases where the kV imager offset increased after maintenance, such as for the Annual PMI and kV detector isocenter calibration, this would indicate that either the calibration performed was less accurate than previously and hence the IsoCal calibration had to work harder to correct, or that the IsoCal calibration itself was inaccurate.

### 4.D Initial beam steering

The MPC tube alignment procedure is based upon measurements taken as part of the MPC geometric checks. The MPC geometric checks are always performed with the 6 MV beam. The center of collimator rotation for the 6 MV beam is used as a spatial reference point for a number of the geometric checks, but the position in space when measured dosimetrically is dependent on the 6 MV beam focal spot alignment with collimator axis. If the beam focal spot is not correctly aligned then the treatment isocenter is affected. It is unclear whether the kV source offset parameter spatially references the 6 MV treatment isocenter, but if so then 6 MV focal spot positon may influence the MPC tube alignment. Focal spot alignment is controlled with beam steering and this is why beam steering was verified prior to performing the MPC tube alignment procedure. Further work could include testing the sensitivity of the MPC kV source offset to changes in 6 MV beam steering. It is hypothesized that the kV source offset would be most sensitive to beam steering in the radial plane, since the radial plane is not in the gantry plane of rotation. Therefore, in this plane the misalignment will not be averaged out with gantry rotation.

### 4.E Quality assurance tests

#### 4.E.1 IsoCal

Since the IsoCal calibration aligns the DICOM coordinates of the images with treatment isocenter then it is possible that any X‐ray tube misalignment could at least partially be corrected for by the IsoCal calibration. Since the maximum IsoCal offset reduced after tube realignment (Table 2) then this suggests that this is true. It would also suggest that the tube was better aligned after realignment, which provides circumstantial evidence to the accuracy of the MPC procedure. It is also conceivable that since IsoCal is required to make smaller corrections to the detector position then the corrections may be more consistent and provide more stable alignment between MV and kV isocenters and better CBCT image quality. The interactions between X‐ray tube alignment and IsoCal and their effect on CBCT image quality and other clinically significant imaging parameters could be investigated more definitively in a further study.

#### 4.E.2 MPC kV Imager offset

The kV imager offset parameter was evaluated by Barnes and Greer^9^ who suggested that the kV imager offset provides a measure of the current IsoCal calibration validity. Since the IsoCal calibration corrects the position of the imager panel position during gantry rotation to help align the imager panel center to treatment isocenter, it was hypothesized that if the tube was misaligned then IsoCal would make adjustments to correct for this within its allowed range. The kV imager offset results of this study appear to support this hypothesis. Before the tube was aligned the kV imager offset was measured at 0.4 mm and near the threshold of 0.5 mm. After tube alignment it was measured at 0.63 mm and out of threshold. This change is attributed to the IsoCal calibration being invalidated by the tube alignment adjustment and this would add evidence to Barnes and Greer's assertion that the kV imager offset provides a measure of IsoCal calibration validity. After the IsoCal calibration was updated the kV imager offset was within threshold to 0.13 mm, which is better than before the alignment procedure. This suggests two things. First, the measured change in kV imager offset of 0.27 mm agrees with the corresponding measured change in IsoCal calibration of 0.37 mm to within 0.1 mm. This provides further evidence that MPC kV imager offset provides a measure of the validity of the IsoCal calibration. Second, the IsoCal procedure does work to correct any tube misalignment, which means that small tube misalignments are likely to be clinically insignificant.

#### 4.E.3 In‐house Winston Lutz

The increase in the left‐right 3D Isocenter offsets in Table 3 after tube realignment suggests that realignment has made the coincidence of treatment and kV isocenter worse. However the standard deviation for this measure over the preceding 12 months is 0.12 mm, which means that the change was less than two standard deviations, indicating that the change is within measurement reproducibility.

#### 4.E.4 CBCT Catphan

A number of CBCT image quality factors as measured with the Catphan phantom were not affected by the tube realignment. This included high contrast resolution, low contrast detectability and spatial integrity. This indicates that either these parameters are either insensitive to a tube alignment change of this magnitude or that the IsoCal calibration is successful at correcting tube misalignment with regards to these parameters. In hindsight, it would've been a good idea to record the IsoCal check procedure results taken before applying a new IsoCal calibration and compare these IsoCal results. This is suggested as part of a future study that investigates more definitively the interaction between IsoCal and X‐ray tube alignment.

The HU values were found to change with the tube realignment, but the change was well within the ±40 HU Varian tolerance and hence considered insignificant. The MPC tube alignment procedure could be updated to include requiring a HU calibration post alignment to ensure accurate HU values. The significant aspect of the Catphan results after the tube realignment is that the image uniformity improved significantly from 17.0 ± 5.6 to 5.0 ± 5.6 (mean ± 2 SD) for full fan and from 10.0 ± 5.6 to 1.0 ± 5.6 for half fan. This improvement is suspected to be due to a reduction in crescent artefact. The crescent artefact can be caused by instability of OBI components due to distortion with gravity with gantry rotation during CBCT. It is possible that with the tube realignment and subsequent reduction in the required IsoCal corrections, the corrections are applied more accurately leading to reduced crescent artefact and better CBCT image uniformity.

#### 4.E.5 OBI Geometric checks and center pixel

The OBI geometric checks and center pixel results showed no significant change after tube realignment. It is suspected that at stationary gantry angles it is relatively easy for IsoCal corrections to be applied correctly and has successfully corrected for the tube misalignment of the magnitude seen in this study.

### 4.F X‐ray tube alignment

#### 4.F.1 Conventional methods

##### Two‐plate method

The two‐plate method results in Fig. 3 show opposing variations in the tangential direction for head up and head down before and after the tube realignment. This indicates that the tube was originally misaligned and after realignment it was misaligned in the opposite direction, which is not in agreement with MPC. Anecdotally the two‐plate method is sensitive to setup inaccuracy. The method requires that the two plates be set up to have precise vertical alignment and this is difficult to achieve using lasers or cross hairs. The method also requires that the gantry be accurately levelled twice (head up and head down), which is another source of uncertainty. Trying to achieve sufficiently high setup accuracy using this method is laborious. These setup uncertainties are a possible explanation for the disagreement with MPC after tube realignment. Also, since the method is performed qualitatively (a weakness in itself) an iterative adjustment to the tube alignment is required which makes the method even more laborious.

##### Wire‐on‐faceplate method

The wire‐on‐faceplate method results of Fig. 4 indicate an increased misalignment in the tangential direction after tube realignment. A possible explanation for this is misalignment of the tungsten wires. Since the wires are placed very close to the X‐ray tube then any slight misalignment in their placement will be magnified at the imager plane and provide an inaccurate result. This is the main weakness of this method. In the axial direction the method is also affected by the kV source isocenter calibration. The kV source isocenter calibration places the kV tube faceplate at a known position relative to linac isocenter, but is not clinically important as long as the tube is correctly aligned. The effect of not performing the isocenter calibration is that the wires attached to the kV source faceplate are no longer aligned correctly between the X‐ray tube and isocenter. This only applies in the axial direction, but is also a potentially prohibitive weakness of the method as it is difficult to perform isocenter calibration with sufficient accuracy.

#### 4.F.2 MPC tube alignment

The MPC tube alignment procedure was quick and simple to perform. Since the method has also been shown to unlikely be affected by phantom setup within clinical significance then it is largely free of setup variation. The MPC procedure reports a measure of tube misalignment as well as the required adjustments. This means that the method is quantitative and allows the user to align the tube with one adjustment. Two adjustments were used in this study, due to confusion about the number of screw turns required from the wizard, but when this was resolved only one adjustment was then required. These advantages are significant over both the two‐plates and wire‐on‐faceplate methods.

The MPC tube alignment procedure could be improved with enhanced clarity of instructions in the wizard. In particular it could be made more specific that the adjustment be made to both of the opposing screws. Also, a sentence stipulating that the number of screw turns to be made relates to after the point where the screws begin to engage would be clearer to the user.

### 4.G Total adjustment time required

The time required for the two‐plates and wire‐on‐faceplate methods are highly variable. Since they are iterative in nature then the number of iterations required will greatly affect the total adjustment time. The experience of the person performing the adjustment, the magnitude of the adjustment and chance will all influence how many adjustment iterations are required and hence how long the procedure will take. The MPC method should not require iterative adjustment and hence it should at least standardize how long an adjustment requires. Based upon the author's experiences the MPC method of tube alignment is significantly quicker to perform than the alternative methods.

## 5 CONCLUSIONS

The MPC method of OBI X‐ray tube alignment was evaluated and compared against the two conventional methods. The three methods were compared for practicality and utility and for sensitivity to a 1 mm realignment of the X‐ray tube as per MPC. The MPC method was found to have high repeatability and to be quick and easy to setup, fast to perform and provide a quantitative measurement with precise tube adjustment instructions. As such, MPC is recommended as the preferred method of X‐ray tube alignment.

The clinical significance of X‐ray tube alignment was also evaluated by performing standard quality assurance tests before and after tube realignment. The quality assurance test results indicated that the magnitude of the tube alignment adjustment in this study was clinically insignificant. This is possibly due to the IsoCal calibration compensating for tube misalignment. However, post tube alignment there was a reduction in the magnitude of the IsoCal correction that was required to be applied and an improvement in CBCT image uniformity. Both results indicate a positive influence of the tube realignment.

## CONFLICT OF INTEREST

The authors disclose that co‐author Buiron Moraro is employed by Varian Medical Systems who are the vendor for the systems evaluated in this study.
